# Fostering Inclusivity in the Clinical Learning Environment

**DOI:** 10.12688/mep.20515.2

**Published:** 2024-10-15

**Authors:** Teresa Y. Smith, Kyla Terhune, Donna A. Caniano

**Affiliations:** 1 Graduate Medical Education and Emergency Medicine, SUNY Downstate Health Sciences University, Brooklyn, New York, 11203, USA; 2 Surgery and Anesthesiology, Vanderbilt University School of Medicine, Nashville, Tennessee, 37235, USA; 3 Surgery, The Ohio State University The Ohio State Health Network, Columbus, Ohio, 43210, USA

**Keywords:** Inclusion, Graduate Medical Education, Inclusivity, Diversity, Equity, and Inclusion, Clinical Learning Enivonrment

## Abstract

Despite the Supreme Court’s decision on race-based admissions, academic medical centers, medical societies, and accreditation bodies remain committed to recruiting a diverse workforce. Many medical schools and graduate medical education programs created initiatives to expand their census of underrepresented in medicine (UIM) as the key to addressing health care disparities. As a result, an influx of an UIM physician workforce has entered clinical learning environments, often without consideration of the inclusivity of these settings. To create inclusive, safe, and comfortable CLEs, we must first recognize the challenges faced by UIM trainees, students, and faculty and the complex ways in which discrimination manifests. Ultimately, having inclusive CLEs allows all learners, especially those from historically excluded identities, to thrive in their training and working environment, making it essential to retain the diverse workforce necessary. Using case examples, we discuss strategies of inclusivity and ways in which we can maintain clinical learning environments where learners feel safe and supported through their training.

## Introduction

The majority of, if not all, academic medical centers and community health systems that sponsor graduate medical education programs, continue to incorporate diversity, equity, and inclusion (DEI) into their mission and aims ^1^. Inclusion refers to the institutional culture that promotes the diversity and uniqueness of each individual through practice, policy, and the development of cultural norms, and creates a high sense of belonging for all members within the organization ^2^. The clinical learning environment (CLE) includes the institutional culture experienced by learners training and working in the clinical setting, and it may vary in the degree to which all learners, including those from historically excluded identities, feel welcomed ^2, 3^. While diversity and equity permit objective measures of success, inclusion relies on how learners and graduate medical education (GME) stakeholders perceive the CLE. Often, the “I” in diversity, equity, and inclusion, is unintentionally underdeveloped in the CLE. In order to create an inclusive CLE that values and respects all members, postgraduate leaders and hospital administrators should consider the formal, informal, and hidden components of training and clinical work which may be inadvertently creating an exclusive and isolating culture.

When an institutional culture is defined by dominant societal norms, learners, including underrepresented in medicine (UIM) trainees, are often expected to acclimate to this culture rather than the institution focusing on creating a culture accepting of differences brought by the diversity of its workforce and patient population ^4, 5^. Achievement of an inclusive CLE requires all learners to feel a sense of belonging that allows them to portray their authentic selves and share their perspective and experiences within the team dynamics in the clinical setting. It is imperative for an inclusive organizational culture to be set with systematic and intentional strategies through the collaboration of the GME and CLE by way of institutional policies, practices, and cultural norms. The following situations illustrate how failure to promote inclusivity may result in an adverse CLE despite the best intentions of graduate medical education leaders. Each situation represents an actual case that has been de-identified to respect confidentiality of participants and clinical sites.

## Case 1

A residency program agreed to host residents in the same specialty from a nearby program for subspecialty rotations unavailable at their clinical site. Residents from the visiting program represented diverse racial and ethnic backgrounds. The host program residents were recruited from the academic institution’s medical school and undergraduate university, and few UIM residents. Within a few months of rotating at the host program, the visiting residents lodged complaints of a discriminatory and unwelcoming CLE at the host program.

When the leadership of both programs and institutions interviewed all the residents, they learned that the residents from the host program, including the few UIM residents, were acclimated to a “common culture” at the host hospital, and comfortable with its CLE. The residents from the host program did not report any experiences of abuse, mistreatment, bias, and/or discrimination. The visiting residents described interactions between themselves and nurses as often confrontational and feeling questioned on their overall medical competency and efficiency. The visiting residents felt excluded from group lunches and did not feel welcomed by the residents of the host program. The visiting residents reported that they had minimal orientation to the host hospital, were not aware of preferred clinical guidelines and medications, and did not have an opportunity to meet the nurses and non-physician staff before assignment to inpatient units. Given the racial and ethnic diversity of the visiting residents, their perception was that their treatment was due to their racial and ethnic backgrounds.

### Strategies of Inclusivity

To welcome the visiting residents the home program should have provided an orientation that included introduction to the host residents, faculty, nurses and other non-physician staff members. The visiting residents should have been given copies of the host program’s clinical guidelines and formulary of preferred medications. Several studies have found that providing rotation-specific orientation (incorporating mobile and/or multimedia to supply site specific information), skills training, and a core lecture series have improved the educational experience of rotating residents ^6– 8^. A buddy system that paired each visiting resident with a host resident as a near-peer mentor may have permitted easier adjustment to the new CLE ^9^.

## Case 2

A fellowship program was excited to accept and train one of their first UIM fellows. Within the first year, the UIM fellow experienced many challenges including judgment based on their accent, given that English was not their first language. While the fellow was successful in completing residency at a prestigious program, they were now experiencing difficulty adjusting to a new CLE where they felt questioned on their medical knowledge and clinical performance. Oftentimes the faculty cited that the fellow had an inability to communicate effectively due to a language barrier. This led to feelings of inferiority and anxiety when presenting patients to the faculty. The fellow avoided speaking up on rounds, which was interpreted as lack of knowledge about patients. The fellow was placed on a performance improvement plan (PIP) and placed at a different teaching site with lower patient volumes, a more diverse patient population, and more intimate interactions with faculty. At this hospital, the fellow began to thrive, and developed a mentoring relationship with a faculty member for whom English was also a second language. The fellow gained confidence, was less afraid to speak up, impressed the faculty at this site with their medical knowledge, and used their bilingual skills as a communication asset. The fellow successfully completed the PIP and graduated with accolades from the program.

### Strategies of Inclusivity

Many UIM trainees find that the “diversity” which they were recruited for, they are later asked to modulate and/or change to acclimate to the dominant societal and working culture ^10^. While addressing concrete deficiencies in academic, clinical, and professional performance are essential, literature has shown that it is equally important to ensure that the CLE is not creating barriers (including macro- and micro-aggressions) which prevent all learners from thriving ^4^. In this case, it was important to utilize the fellow’s diverse language skills as a positive attribute. It has been shown that doctor patient language concordance is beneficial to the quality of patient care, and thus is an asset ^11^. In addition, early mentorship is essential. While mentorship is important for all trainees, it is fundamental in the retention of UIM trainees. An example is Indiana University’s Incentivizing Diverse Recruitment for Equity in Academic Medicine (iDREAM) program which offers mentorship and stipends for UIM residents who commit to faculty positions at the academic center upon graduation. This mentorship also ensures bidirectional feedback about resident performance and the clinical learning environment’s acceptability and continuous evaluation to ensure it is free of bias ^12, 13^.

## Case 3

A second-year resident was assigned to a high acuity service following an eight-week parental leave of absence. The resident experienced challenges upon returning to work, including time and availability of convenient locations for lactation. The lactation space available in the hospital was distant from clinical activities, and did not have a computer, therefore, the resident was unable to complete patient care notes while pumping. This resulted in a state of anxiety about missing clinical duties and pending work. Ultimately, the resident had poor milk production and discontinued human milk feeding. During the semiannual evaluation the resident received negative comments about absences from clinical duties since returning from parental leave. The resident did not feel free to discuss with the program director the challenges with lactation for fear of retaliation and gender discrimination.

### Strategies of Inclusivity

The CLE must have lactation accommodations that are close to patient care units and fitted with computers that permit residents to continue with clinical duties while pumping (Accreditaton Council of Graduate Medical Education (ACGME) Institutional Requirement III.B.7.d).(4)) ^14^. Program leadership should have considered assigning the resident to a lower acuity inpatient service or an outpatient selective for the first rotation following parental leave. The resident should be provided with mentorship and a means to give feedback on how she is adjusting to the return from parental leave, and the dual roles of mother and physician-in-training ^15^. Programs have successfully implemented inclusion practices, such as flexible scheduling for pregnant and new parent residents that have been accepted by trainees and added no additional burden to scheduling clinical work ^16^.

## Discussion

Creation of inclusive, safe, and comfortable CLEs requires recognition of the challenges faced by all trainees, and the complex ways in which exclusion manifests, especially for UIM residents ^17^. These challenges are frequently set by entrenched societal discriminations, biases, and gendered treatment that underlies the original foundation of medical training ^4, 5^. Ultimately, having inclusive CLEs allows all learners to thrive in their training and working environment. Program, GME, and CLE leaders must be proactive in the intentional design of inclusive practices in conjunction with their diversity and equity initiatives. Faculty development is essential for faculty to receive up-to-date, practical diversity, equity, inclusion, and belonging training that prepares them to work with a diverse group of learners. Programs should consider faculty mentorship and/or peer mentorship programs. Program leaders should encourage bidirectional feedback with frequent check-in meetings with trainees to build trust so that when concerns are brought forth, there will be every attempt to address them. Ongoing dialogue between program leaders, learners, and other members of the clinical team permit all stakeholders to co-create the inclusive attributes and monitoring of their CLE.

Several programs have published toolkits which can be used as a framework that describe their successful strategies to advancing DEI in graduate medical education, including the ACGME’s Equity Matters initiative ^18, 19^. The ACGME launched the Barabara Ross-Lee DO DEI Award which recognizes intuitions who have successfully implemented DEI efforts across their GME programs ^20^. In an exploratory content analysis of the award applications, Boatright *et al.* describe successful inclusion practices such as DEI committees, faculty/resident-led affinity groups, longitudinal DEI curricula, and community partnerships ^21^. Some of the themes of success are institutional support, an invested community of stakeholders, and a shared value among all learners, program and institutional leaders, and other members of the team within the CLE. The challenges are centered around the institutional buy-in and the equal prioritization of both GME and CLE, which includes providing funding and resources towards success of the DEI initiatives ^18^. Organizational culture change is the strongest barrier to success of any change management initiative, and thus using intentional strategies to set new cultural norms takes a collaborative effort of the programs, GME, and CLE. There must be a commitment to go beyond appreciation of DEI to actually incorporating these efforts into the fabric of the organizational culture of the CLE and its training programs ^22^. Our discussion is limited in scope, and just touches the surface of the breadth of innovation into the area of DEI, specifically inclusion. The key to creating inclusive clinical learning environments for all learners is developing a clear focus on the “I” in DEI. 

## Data Availability

No data are associated with this article.
