# Supplementary material for: Fostering Inclusivity in the Clinical Learning Environment
Source: MedEdPublish (2016). 2024 Oct 15;14:61. Originally published 2024 Aug 9. [Version 2] doi: 10.12688/mep.20515.2 (PMC11490830; doi:10.12688/mep.20515.2)
Supplement: Peer review history

# Reviewer report: Reviewer response for version 2

**DOI:** 10.21956/mep.22198.r39553

**Published:** 2024-11-15

**Authors:** Brett R Vaughan

**Affiliations:** 1 Department of Medical Education, The University of Melbourne, Melbourne, VIC, Australia

Thank you for the opportunity to review this interesting work.  The work has the potential to inform strategies to improve and foster inclusivity in clinical learning environments.  At present, the article is somewhat North American-centric.  For example, the first line of the abstract refers to the a Supreme Court decision.  This assumes the reader is aware of the ruling.  Some of the language also reflects the North American-centricity of the work.  It may be useful to review the work in this light and either highlight these strategies from a North American perspective, or broaden the language to enable the strategies to be applied to other jurisdictions.  The three cases are quite good and well supported by relevant literature.  There could be modifications to the work to highlight how some of these issues could be addressed in more resource-constrained environments too if the work is taken beyond the North Armerican perspective.

If evidence from practice is presented, are all the underlying source data available to ensure full reproducibility?

Yes

Is the topic of the practical tips discussed accurately in the context of the current literature

Partly

Are all factual statements correct and adequately supported by citations?

Yes

Are the conclusions drawn balanced and justified on the basis of the presented arguments?

Yes

Are arguments sufficiently supported by evidence from the published literature and/or the authors’ practice?

Yes

Reviewer Expertise:

Clinical learning environments

I confirm that I have read this submission and believe that I have an appropriate level of expertise to confirm that it is of an acceptable scientific standard, however I have significant reservations, as outlined above.

# Reviewer report: Reviewer response for version 2

**DOI:** 10.21956/mep.22198.r39350

**Published:** 2024-11-04

**Authors:** Russyan Mark Mabeza, Akeive Burrows

**Affiliations:** 1 University of California San Francisco, San Francisco, California, USA; 2 University of California Los Angeles David Geffen School of Medicine (Ringgold ID: 12222), Los Angeles, California, USA

The authorship team did an excellent job addressing the comments from our prior review. We are pleased to offer an Approval Status for indexing of this manuscript.

If evidence from practice is presented, are all the underlying source data available to ensure full reproducibility?

Not applicable

Is the topic of the practical tips discussed accurately in the context of the current literature

Yes

Are all factual statements correct and adequately supported by citations?

Partly

Are the conclusions drawn balanced and justified on the basis of the presented arguments?

Partly

Are arguments sufficiently supported by evidence from the published literature and/or the authors’ practice?

Partly

Reviewer Expertise:

Antiracism, medical education

We confirm that we have read this submission and believe that we have an appropriate level of expertise to confirm that it is of an acceptable scientific standard.

# Reviewer report: Reviewer response for version 2

**DOI:** 10.21956/mep.22198.r38759

**Published:** 2024-10-19

**Authors:** Kat Butler

**Affiliations:** 1 University of Toronto, Toronto, Canada

**Fostering Inclusivity in the Clinical Learning Environment**

**Overall**

 This is a helpful paper which provides realistic case studies and concrete tips for programs that may face similar issues, or issues stemming from similar underlying inequity in clinical learning environments. 

 My only question, which is one I have whenever the topic of inclusion and specifically ‘including all’ is brought up, is to consider who we mean to include when we say inclusive environments will allow “all learners” to thrive - the authors here specify ‘especially those from historically excluded identities,’ and their meaning is clear. But do we also want to have people who hold or promote vehemently racist, sexist, homophobic, ableist, transphobic or otherwise oppressive beliefs to thrive in our learning environments ? Of course, as the authors point out, CLEs unfortunately have been established with these forms of discrimination embedded in them.

 Overall I do believe that CLEs which take into account the wide range of human needs and cultures (and the varying experiences, needs, and strengths of learners as individuals) truly benefit learners from all groups, and also that these spaces may not always feel ‘safe’ as we challenge one another to grow and question our underlying beliefs and long-held institutional practices. 

**Abstract**

 Briefly situates the topic in the current American legal context; given that this is an international journal it may be helpful to specify to which Supreme Court the authors are referring.  I also note that this context is not included in the introduction, but only in the abstract. Otherwise an excellent summary of the paper.

**Introduction**

 Again, a helpful and brief summary of the issue at hand and the knock-on effects of an overemphasis on recruitment without a simultaneous focus on changing the culture in clinical learning environments. A further citation that may be helpful for the sentence about the importance an environment that supports learners in feeling a sense of belonging may be the Sternszus R et al.(2024 ^1^ ) paper I have included a link to below. 

**Cases**

 Excellent cases which certainly resonate with challenges faced by programs and residents. I did find it interesting that the authors used gender neutral language about the resident in case 3 in the case description, but then used gendered pronouns in the strategies for inclusivity. I don’t think that this detracts from the paper in any way, just noticed the shift between the two sections.

**Strategies of Inclusivity**

 Concrete and relevant tips for addressing these issues. 

**Discussion**

 The discussion moves appropriately to higher level guidance for considerations of improving inclusivity in PG programs. The emphasis on faculty education is certainly one that is very supported by literature and practice.

If evidence from practice is presented, are all the underlying source data available to ensure full reproducibility?

Not applicable

Is the topic of the practical tips discussed accurately in the context of the current literature

Yes

Are all factual statements correct and adequately supported by citations?

Yes

Are the conclusions drawn balanced and justified on the basis of the presented arguments?

Yes

Are arguments sufficiently supported by evidence from the published literature and/or the authors’ practice?

Yes

Reviewer Expertise:

Trans inclusion in medical education

I confirm that I have read this submission and believe that I have an appropriate level of expertise to confirm that it is of an acceptable scientific standard.

## References

[1] : Being, becoming, and belonging: reconceptualizing professional identity formation in medicine. *Front Med (Lausanne)* .2024;11: 10.3389/fmed.2024.1438082 1438082 10.3389/fmed.2024.1438082 39257893 PMC11383779

# Reviewer report: Reviewer response for version 1

**DOI:** 10.21956/mep.21957.r38761

**Published:** 2024-10-01

**Authors:** Russyan Mark Mabeza, Akeive Burrows

**Affiliations:** 1 University of California San Francisco, San Francisco, California, USA; 2 University of California Los Angeles David Geffen School of Medicine (Ringgold ID: 12222), Los Angeles, California, USA

Thank you for the opportunity to review this article. In brief, this manuscript emphasizes the importance of integrating and tailoring specific needs into diversity, equity, and inclusion (DEI) efforts within graduate medical education programs, particularly the clinical learning environment (CLE). The article presents three cases which demonstrate how a CLE’s failure to effectively promote inclusivity can negatively impact trainees that are underrepresented in medicine (UIM). For each case, it offers strategies for improvement and advocates for proactive efforts by educational leadership to design inclusive practices centered around UIM trainee needs, alongside diversity and equity initiatives.

 This is important work and can be useful for graduate medical education programs who are aiming to bolster inclusion initiatives within their institutions.

 Please consider the following comments upon revision of the article:

  

- Consider providing a more explicit statement to establish the necessity for systematic implementation of inclusion strategies in graduate medical education. 

 

- The case studies provide clear and concrete illustrations of instances of exclusion. They effectively highlight real-life consequences of a CLE that is not inclusive for UIM trainees.

 

- The cited strategies are clear, practical, and actionable. However, they lack evidence of effectiveness or critical analysis of potential promise and limitation. 

 

- Consider providing an operational framework for how suggested strategies might be implemented. It would be helpful to cite prior studies that demonstrate in some detail how these strategies could look like. If not from prior studies, consider providing perspectives from authors’ own experiences of operationalizing these strategies in their own institutions. 

 

- Consider discussing the challenges and other factors that programs should consider when  attempting to implement these strategies, including differences in institutions, political systems within which GME programs exist, financial/logistical constraints, etc. 

 

- Consider discussing the limitations inherent to this article.

 

- Consider revising the first sentence, as it is not necessarily supported by the first citation.

If evidence from practice is presented, are all the underlying source data available to ensure full reproducibility?

Not applicable

Is the topic of the practical tips discussed accurately in the context of the current literature

Yes

Are all factual statements correct and adequately supported by citations?

Partly

Are the conclusions drawn balanced and justified on the basis of the presented arguments?

Partly

Are arguments sufficiently supported by evidence from the published literature and/or the authors’ practice?

Partly

Reviewer Expertise:

Antiracism, medical education

We confirm that we have read this submission and believe that we have an appropriate level of expertise to confirm that it is of an acceptable scientific standard, however we have significant reservations, as outlined above.

# Reviewer report: Reviewer response for version 1

**DOI:** 10.21956/mep.21957.r38768

**Published:** 2024-09-30

**Authors:** Arlene Chung

**Affiliations:** 1 University of Vermont Health Network, Burlington, Vermont, USA

This is a brief report that provides an overview of the literature to date regarding inclusion in the clinical learning environment, followed by three real-life cases that have been de-identified to respect the confidentiality of the participants and the clinical sites. The cases are meant to illustrate common challenges and provide practical strategies for addressing them. While the literature on diversity, equity, and inclusion is still evolving, the authors provide adequate and factual background on the topic and best practices known to date. The report is well written and the authors’ arguments are logical and justified. Overall, I recommend indexing for this publication.

If evidence from practice is presented, are all the underlying source data available to ensure full reproducibility?

Not applicable

Is the topic of the practical tips discussed accurately in the context of the current literature

Yes

Are all factual statements correct and adequately supported by citations?

Yes

Are the conclusions drawn balanced and justified on the basis of the presented arguments?

Yes

Are arguments sufficiently supported by evidence from the published literature and/or the authors’ practice?

Yes

Reviewer Expertise:

Medical education; graduate medical education; well-being; mentorship; medical education research

I confirm that I have read this submission and believe that I have an appropriate level of expertise to confirm that it is of an acceptable scientific standard.
